# The embryonic life history of the tropical sea hare *Stylocheilus striatus* (Gastropoda: Opisthobranchia) under ambient and elevated ocean temperatures

**DOI:** 10.7717/peerj.2956

**Published:** 2017-02-01

**Authors:** Rael Horwitz, Matthew D. Jackson, Suzanne C. Mills

**Affiliations:** 1 Paris Sciences et Lettres (PSL) Research University: École Pratique des Hautes Études (EPHE)-Université de Perpignan Via Domitia (UPVD)-Centre National de la Recherche Scientifique (CNRS), Unité de Service et de Recherche 3278 Centre de Recherches Insulaires et Observatoire de l’Environnement (CRIOBE), Papetoai, Moorea, French Polynesia; 2 Laboratoire d’Excellence “CORAIL”, Moorea, French Polynesia; 3 School of Geography and Environmental Sciences, Ulster University, Coleraine, UK

**Keywords:** Opisthobranch, Embryonic development, Ocean warming, Life history, Sea hare, Elevated temperature, *Stylocheilus striatus*

## Abstract

Ocean warming represents a major threat to marine biota worldwide, and forecasting ecological ramifications is a high priority as atmospheric carbon dioxide (CO_2_) emissions continue to rise. Fitness of marine species relies critically on early developmental and reproductive stages, but their sensitivity to environmental stressors may be a bottleneck in future warming oceans. The present study focuses on the tropical sea hare, *Stylocheilus striatus* (Gastropoda: Opisthobranchia), a common species found throughout the Indo-West Pacific and Atlantic Oceans. Its ecological importance is well-established, particularly as a specialist grazer of the toxic cyanobacterium, *Lyngbya majuscula*. Although many aspects of its biology and ecology are well-known, description of its early developmental stages is lacking. First, a detailed account of this species’ life history is described, including reproductive behavior, egg mass characteristics and embryonic development phases. Key developmental features are then compared between embryos developed in present-day (ambient) and predicted end-of-century elevated ocean temperatures (+3 °C). Results showed developmental stages of embryos reared at ambient temperature were typical of other opisthobranch species, with hatching of planktotrophic veligers occurring 4.5 days post-oviposition. However, development times significantly decreased under elevated temperature, with key embryonic features such as the velum, statocysts, operculum, eyespots and protoconch developing approximately 24 h earlier when compared to ambient temperature. Although veligers hatched one day earlier under elevated temperature, their shell size decreased by approximately 20%. Our findings highlight how an elevated thermal environment accelerates planktotrophic development of this important benthic invertebrate, possibly at the cost of reducing fitness and increasing mortality.

## Introduction

Increasing anthropogenic carbon dioxide (CO_2_) emissions are changing ocean conditions at an accelerating rate (Hoegh-Guldberg & Bruno, 2010). Elevated sea surface temperature (SST) is accompanied by increased partial pressure of CO_2_ (*p*CO_2_) in the ocean, which changes the relative amounts of inorganic carbon, ultimately making the ocean more acidic (Gattuso et al., 2015). The cumulative effects of these stressors on marine biota may be detrimental for many taxa, with important consequences for early life-history traits (Beckerman et al., 2002). Environmental conditions experienced during these initial developmental stages, including elevated seawater temperature (e.g., Byrne et al., 2009), decreased pH (e.g., Kurihara, 2008) and low oxygen (e.g., Gobler et al., 2014), can have profound effects on the subsequent performance of individuals. Thus, evaluation of environmental change impacts on organism reproductive success, growth and physiology dictates that their most sensitive life cycle stages must first be characterized.

**Figure 1:**
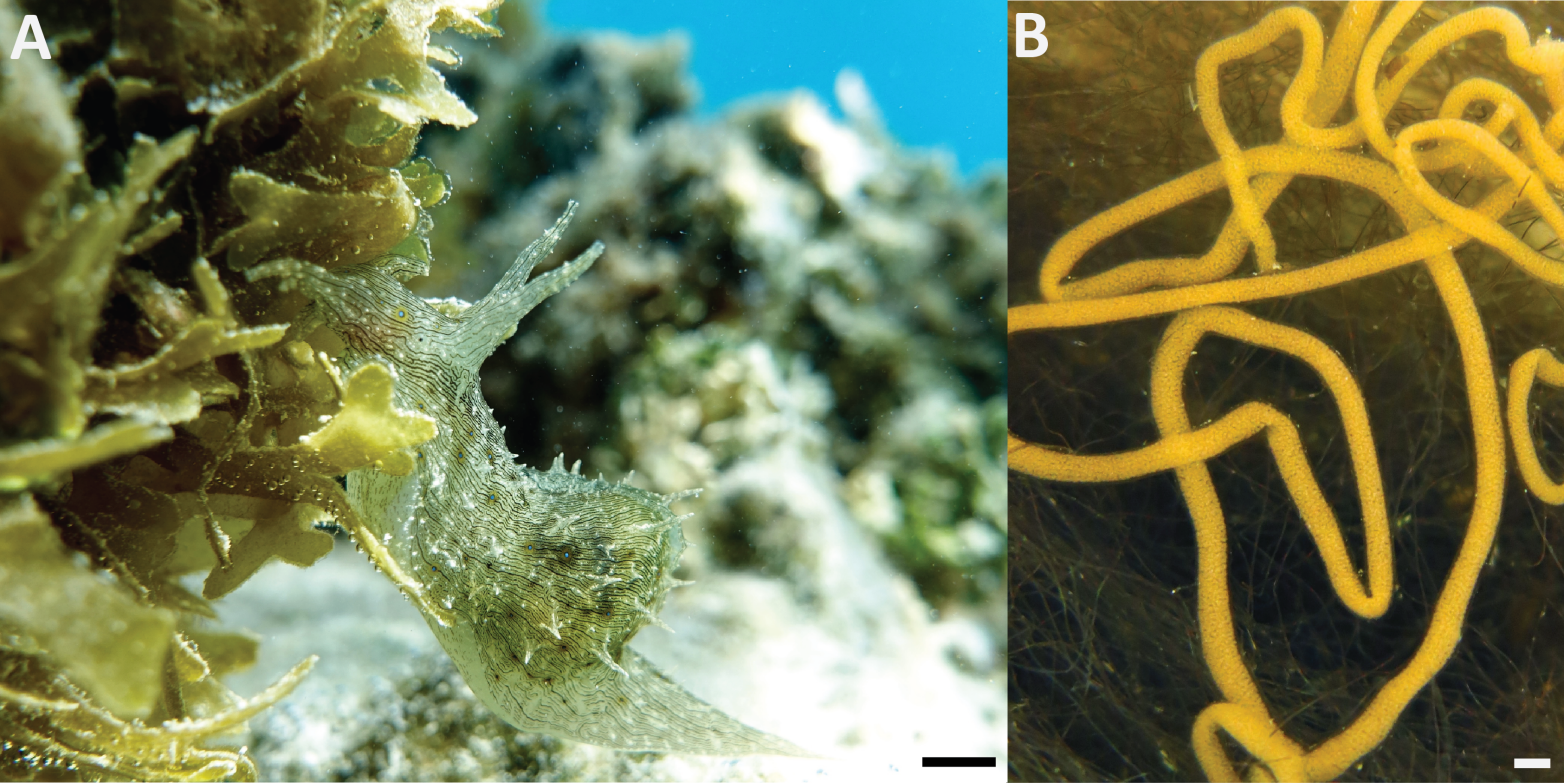
(A) *Stylocheilus striatus*. Scale bar = 5 mm. Photograph by SCM. (B) Typical egg ribbon of *S. striatus* laid in the toxic cyanobacterium, *Lyngbya majuscula*. Photograph by RH. Scale bar = 1 mm.

The opisthobranch sea hare *Stylocheilus striatus* (Quoy and Gaimard, 1832) is a circumtropical species found throughout the Indo-West Pacific and Atlantic Oceans (Thompson, 1976) (Fig. 1A). This species is relatively well-studied, including many aspects of their taxonomy (Bebbington, 1974), anatomy (Bebbington, 1977), physiology (Switzer-Dunlap, 1978; Carefoot, 1987), reproductive patterns (Switzer-Dunlap & Hadfield, 1979), diet-derived chemical defense (e.g., Paul & Pennings, 1991; Nagle, Camacho & Paul, 1998; Arthur et al., 2009) and sensitivity to noise pollution (Nedelec et al., 2014). The important role they play in benthic reef ecology is well-established, particularly as a specialist grazer of the toxic cyanobacterium, *Lyngbya majuscula* (Thacker & Paul, 2004), that prevents the settlement of coral larvae (Kuffner & Paul, 2004) and can cause phase shifts from coral to algal dominated reefs (Thacker, Ginsburg & Paul, 2001). However, their embryonic developmental life history has not been investigated and there is a lack of basic information about their initial life stages (but see EJ Armstrong, T Allen, M Beltrand, V Dubousquet, JH Stillman, SC Mills, 2012 & 2015, unpublished data).

*Stylocheilus striatus* are hermaphroditic and exchange sperm by copulation shortly before egg-laying (oviposition), sometimes forming mating chains of three or more individuals to increase fertilization efficiency (Switzer-Dunlap & Hadfield, 1981). Eggs are laid in gelatinous strings (Fig. 1B), in which the eggs are segregated in capsules, eventually hatching as planktotrophic veliger larvae. Their calcareous shell (i.e., protoconch) is maintained only during the pelagic larval stage prior to settlement and metamorphosis.

This study provides an account of copulation, oviposition and the embryonic life history of *S. striatus*, thus adding essential information to the biology and ecology of this important benthic invertebrate. Additionally, the effects of elevated seawater temperature, associated with climate change, on developmental time of key embryonic features are determined by comparison between embryos developed in present-day and predicted end-of-century ocean temperatures (+3 °C from present-day, consistent with the Representative Concentration Pathway (RCP) 8.5 scenario of the Intergovernmental Panel on Climate Change (IPCC) for 2100 (Intergovernmental Panel on Climate Change, 2013)).

## Materials and Methods

### Study site

Adult specimens of *S. striatus* were collected from the lagoon of Mo’orea Island (French Polynesia, Pacific Ocean; 149°50′W, 17°30′S), at depths of 1–15 m on flat reefs within blooms of the cyanobacteria, *L. majuscula*. Approval was granted from the institutional animal ethics committee at the CRIOBE research station for specimen collection and dissection (permit Number: 006725).

### Husbandry, broodstock characteristics, reproductive behavior and oviposition

After collection, sea hares were kept in aquaria (40-L) at CRIOBE and supplied with oxygenated running seawater (0.5 l min^−1^) at ambient light and temperature regimes. Ambient seawater temperature was in the range of 27.7–28.5 °C and salinity was 35–36.3 psu. Following a three-week acclimation period, the whole-body length of each sea hare was measured, individuals were isolated in individual 15 × 5 × 5 cm plastic breeding containers for 12 h, after which similarly sized individuals (<1 cm difference) were paired in the breeding containers (*n* = 21 pairs). Pairs were monitored every 20 min until they were observed copulating. The percentage of simultaneously reciprocal mating (i.e., reciprocal penis intromissions, insemination and subsequent egg-laying) (*n* = 21 pairs) were recorded, as were other courtship and copulation behaviors (e.g., total duration and position) (*n* = 5 pairs). Once the pair was observed having separated from the copulation position, individuals were returned to individual breeding containers to prevent any further mating. Time to oviposition (*n* = 12) and its duration (*n* = 5) were also recorded. Differences in sample sizes from the initial *n* = 21 were due to whether observations were logistically possible, for example if copulation and/or oviposition occurred at night and whether eggs were found within 1 h of oviposition.

### Egg mass characteristics

As soon as oviposition had occurred, egg ribbons were taken from twelve mothers and morphological descriptors of the egg ribbons (*n* = 12), such as shape, color and rotation form, were determined as described by Wilson (2002). After measuring the total length and width of the egg ribbons, the density of embryos (number of embryos/mm^2^ egg ribbon; *n* = 10 mm^2^ areas per egg ribbon) and number of embryos per egg capsule were counted (*n* = 10 egg capsules per egg ribbon) using a light stereomicroscope, Nikon SMZ2100, coupled to a Motic Image digital camera and Motic Image Plus Software. Caution was used to count egg capsules from the middle of the ribbon as well as from the edges.

### Embryonic development

Nine egg ribbons were taken from nine mothers who had been observed laying eggs and development until hatching was monitored under ambient temperature. Egg ribbons were cut into two equally sized sections with a scalpel and split randomly between two replicate aquaria (10-L aquaria) under ambient (28 °C) temperature. The diameter of uncleaved embryos was measured from random samples (*n* = 5 embryos per cut section; *n* = 10 embryos per egg ribbon; *n* = 90 embryos in total) using an ocular micrometer. Each developmental stage was described, measured and photographed every hour for the first 24 h post-oviposition, and then every 2 h until hatching (*n* = 4–5 photos per cut section; *n* = 20 embryos per cut section; *n* = 360 embryos in total). A new stage of development was considered attained when it was reached by ∼50% of the embryos (following Hamel, Sargent & Mercier, 2008). The progress of embryonic development was monitored by identifying the 10 stages proposed by Buckland-Nicks, Gibson & Koss (2006): oviposition, 1st cleavage, 2nd cleavage, 3rd cleavage, morula, blastula, gastrula, movement by cilia, trocophore and early pre-hatching embryos. Data on time to reach each developmental stage was standardized as the time post-oviposition. Time to hatching (h) was also measured. Shell patterns of the early veliger stage were categorized according to Thompson (1961).

### Effects of temperature on key embryonic features

Five separate egg ribbons were taken from five mothers who had been observed laying eggs and were cut into six equally sized sections with a scalpel and split randomly between three replicate ambient (28 °C) and three replicate elevated temperature (+3 °C; 31 °C) treatments (representing upper-threshold for the IPCC “business-as-usual” scenario (RCP 8.5) for 2100 (Intergovernmental Panel on Climate Change, 2013); the RCP 8.5 scenario in this study refers only to temperature predictions and not added pH changes) (*n* = 5 egg ribbons, each with three replicate cut sections per treatment). Cut sections in the elevated temperature treatment were ramped from 28 °C to 31 °C over 3 days (1 °C/1 d). Egg ribbon sections were photographed every hour for the first 24 h post-oviposition, and then every 2 h until hatching. The following embryonic features were recorded from 3–4 photographs of 20 embryos per cut section and compared between temperature treatments: velum, statocyst, left and right digestive diverticula, operculum, eyespots and protoconch (based on observations described in Hadfield & Switzer-Dunlap, 1984). Lateral shell length and width (spiral height) of newly hatched veligers were measured randomly from the cut sections (*n* = 6 veligers per egg ribbon in each treatment; *n* = 30 veligers per treatment; *n* = 60 veligers in total). Differences in development time were tested for significance using a mixed-model nested analysis of variance (ANOVA) with embryonic feature (e.g., velum) as the dependent variable, treatment as the independent (fixed) variable and replicate nested within ribbon as a random variable. Paired *t*-tests were used to test for differences in shell dimensions of newly hatched veligers between ambient and elevated temperature treatments. Results were considered significant for a *p* value <0.05. All data were analyzed using R (R Core Team, 2016).

## Results

### Broodstock characteristics, reproductive behavior and oviposition

The mean body length of the *S. striatus* broodstock was 5.6 ± 0.73 cm (*n* = 12) (see Table 1). Individuals were generally observed initiating multiple contacts before exchanging sperm during copulation with their partners, aligning head-to-tail with right sides opposing, to enable simultaneous sperm transfer between the pair (the genital opening is on the right side of the head), with the percentage of simultaneously reciprocal mating at 47%. The total copulatory duration ranged from 15 to 60 min, while time to oviposition varied from 5 to 16 h. Duration of oviposition had an average time of 9.8 ± 3.56 min (*n* = 5). All data are summarized in Table 1.

**Table 1:** Biology and reproductive behavior of the tropical sea hare, *Stylocheilus striatus*.

	Mean (±SD)
Length of adult individuals (cm) (*n* = 12)	5.6 (0.73)
Total copulatory duration (min) (*n* = 5)	41 (16.35)
Time to oviposition post-copulation (h) (*n* = 12)	9.2 (3.42)
Oviposition duration (min) (*n* = 5)	9.8 (3.56)
Simultaneously reciprocal mating (%) (*n* = 21 pairs)	47

### Egg mass characteristics

The eggs produced by *S. striatus* are extruded as a ribbon-shaped mass, and can be described as being type A opisthobranch (Hurst, 1967) (Fig. 1B). The egg ribbon consisted of embyros in egg capsules (i.e., thin membrane structure) embedded in a jelly-like coating layer, which was adhered to the substratum during oviposition. In addition, the egg ribbon was curved, twisting and overlapping back on itself numerous times, but at all times attached to the substratum (or itself) (Fig. 1B). The length of an egg ribbon ranged between 11.5 and 30 cm (see Table 2), whereas their width was approximately 1 mm. The mean number of embryos per mm^2^ of egg mass (±SD) was 100 ± 13.96, while a typical egg capsule contained an average of 2.68 ± 0.76 embryos within a range of 2–5 (Table 2). When first laid, the ribbon often had a pale yellowish color, but with subsequent development they darkened, changing to brown.

**Table 2:** Egg mass characteristics of *Stylocheilus striatus* (*n*= 12).

Variable	Mean (±SD)
Egg mass length (mm)	19.81 (5.75)
No. of embryos/mm^2^ egg mass	100 (13.96)
No. of embryos/egg capsule	2.68 (0.76), range 2–5
Diameter of uncleaved embryo (µm)	60.84 (2.55)

### Embyronic development

**Figure 2:**
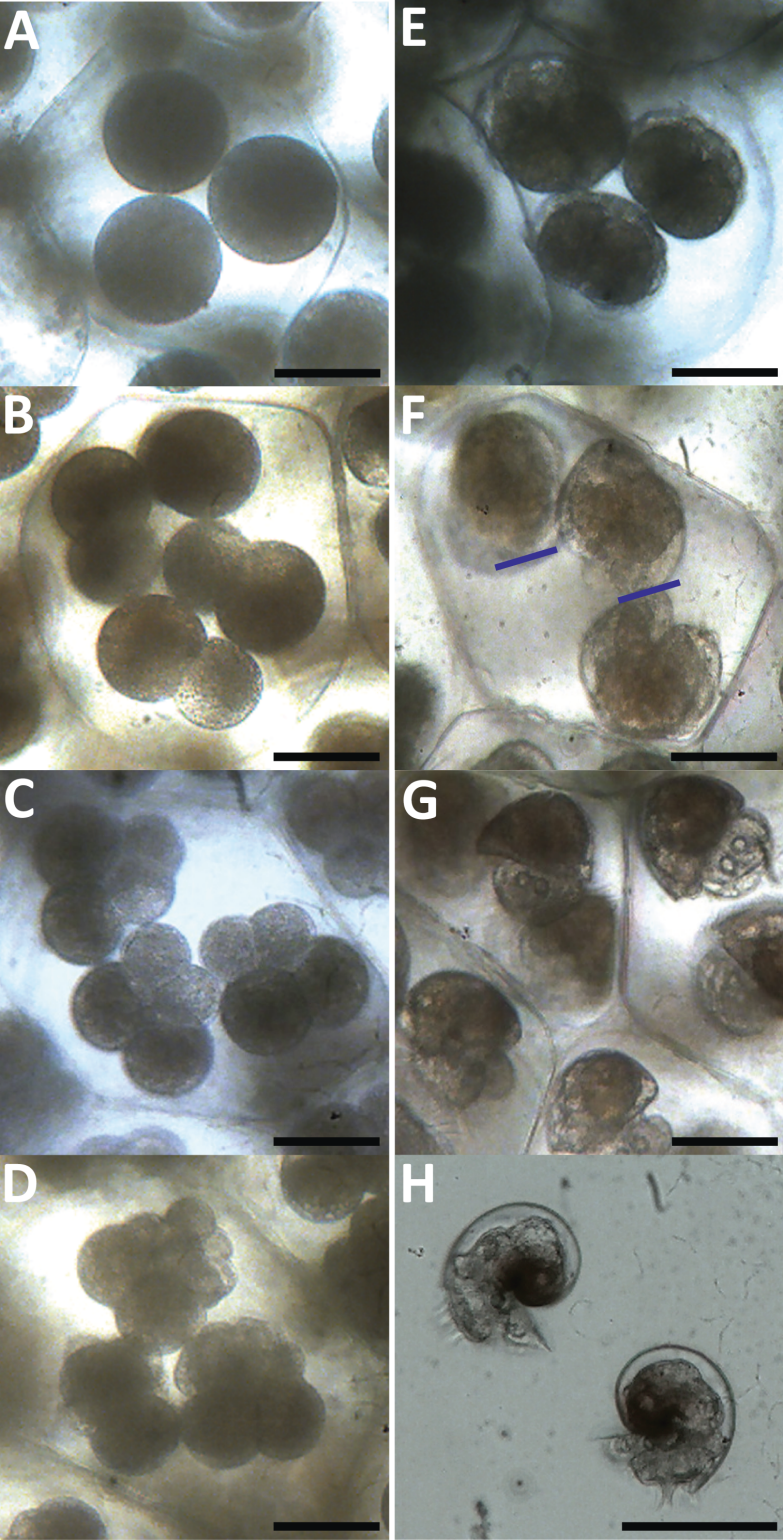
Development of *Stylocheilus striatus* within the egg ribbon. (A) Uncleaved embryos within an egg capsule (0–1 h post-oviposition). (B) First cleavage (3 h post-oviposition). (C) Second cleavage (5 h post-oviposition). (D) Third cleavage (6.5 h post-oviposition). (E) Morula stage (16.5 h post-oviposition). (F) Trocophore (57 h post-oviposition): embryo elongate, blue lines indicate the bottom end of the top two embryos where the pedal rudiment and velar field are present. (G) Three-day-old pre-hatching larvae showing developed velum and statocysts. (H) Young juvenile veligers 1 day post-hatching, showing larval shell complete, left and right digestive diverticulum. Scale bars for A–G = 50 µm; H = 100 µm.

Uncleaved embryos had an average diameter (±SD) of 60.84 (±2.55) µm (Fig. 2A). Table 3 is a summary of the average time of embryonic developmental stages under ambient temperature. The first cell division (1st cleavage) in the early embryo occurred within 2–4 h after oviposition, resulting in two equally-sized cells resembling a “figure of 8” (Fig. 2B). The 2nd cleavage occurred 4–6 h after oviposition forming four cells converging in the middle (Fig. 2C). At this stage the two new cells had a lighter color than the original cells, most likely as a result of asymmetrical distribution of the egg cytoplasm (Page, 2007). At the 8-cell stage (3rd cleavage), occurring within 6–8 h, the cells from the first cleavage retained their dark color and the new cells were again light and clear, but even smaller than the new cells formed from the 2nd cleavage (Fig. 2D). The morula stage appeared between 16–17 h after oviposition, which was characterized by the cytoplasm evidently spreading from the original two cells towards the centre of the embryo (Fig. 2E). The blastula stage followed at 26–30 h post-oviposition, while gastrulation took place within a range of 38–44 h, creating the characteristic heart-shape. Movement gradually became evident at this stage as cilia began to form on the velar rudiment (evident at 42–46 h). Thereafter, they developed to the trocophore stage at 54–62 h post-oviposition (Fig. 2F). The bottom and top ends of the heart-shape began to elongate from one side, however this was more obvious for the bottom end (see blue lines in Fig. 2F). The bottom half later transformed into features such as the velum, operculum and statocysts, whilst the top half (where the cytoplasm remained) later developed into the protoconch and eye spots. At this stage rotation became increasingly evident due to the velum becoming more profound and the cilia improving circulation within the egg capsules. Features of pre-hatching embryos continued to develop 3 days post-oviposition and could be found in the cytoplasm which began to retract further into the protoconch (Fig. 2G).

**Table 3:** Embryonic developmental stages of *Stylocheilus striatus* within the egg capsule under ambient temperature. Mean time is measured in hours (h) and days (d) (±SD), as well as range. Data were compiled from the observation of 9 different egg masses.

Stage of development	Mean time (±SD), range
Oviposition (Fig. 2A)	0 h
1st cleavage (Fig. 2B)	2.8 h (0.6), 2–4 h
2nd cleavage (Fig. 2C)	4.7 h (0.83), 4–6 h
3rd cleavage (Fig. 2D)	6.7 h (0.66), 6–8 h
Morula (Fig. 2E)	16.4 h (0.52), 16–17 h
Blastula	27.7 h (1.2), 26–30 h
Gastrula	41.3 h (1.73), 38–44 h
Cilia movement	44.8 h (1.45), 42–46 h
Trocophore (Fig. 2F)	58.4 h (2.78), 54–62 h
Pre-hatching larvae (Fig. 2G)	3.1 d (0.08), 70–80 h
Hatching	4.5 d (0.2), 4.08–4.75 d

### Hatching

After 70 to 80 h the embryos developed into early-stage veligers with increased rotation. The capsules restricted embryonic movement and they slowly began to deteriorate and eventually broke apart, thus enabling the young veligers to hatch. Hatching occurred between 4.08–4.75 d after egg-laying. Veligers began to hatch randomly along the length of the egg mass, with the newly hatched larvae moving around rapidly, thus deteriorating the condition of the adjacent egg capsules and causing them to hatch as well. Hatching then spread along the ribbon in both directions and the larvae broke through the egg ribbon wall. All features of the newly hatched veligers were fully developed and functional. The thin and transparent larval shells of *S. striatus* can be described as type 1, following Thompson (1961). Organs and structures were visible through the shell and the cytoplasm was fully retracted to its far end (Fig. 2H).

### Effects of temperature on key embryonic features

**Figure 3:**
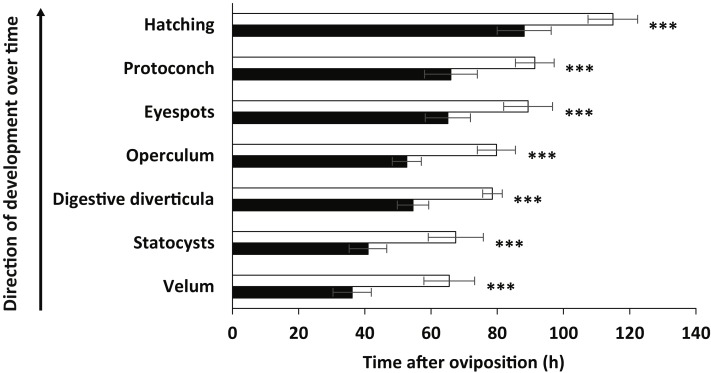
Key visible events of embryonic development of *Stylocheilus striatus*. White and black bars indicate ambient and elevated (+3 °C) temperature conditions, respectively. *Asterisks* show significant differences from ambient temperature (mixed-model nested ANOVA, ^∗∗∗^ = *p* < 0.001). Error bars show means ± SD.

**Figure 4:**
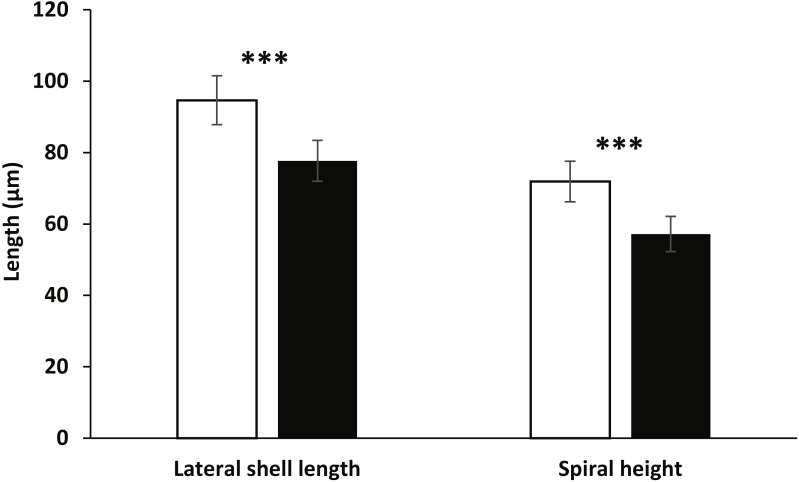
Veliger shell dimensions. White and black bars indicate ambient and elevated (+3 °C) temperature conditions, respectively. *Asterisks* show significant differences from ambient temperature (paired *t*-test, ^∗∗∗^ = *p* < 0.001). Error bars show means ± SD.

The developmental stages were the same as described above for embryos in ambient seawater temperature, but the relative timing to reach each stage was significantly more rapid in the elevated temperature conditions (mixed-model nested ANOVA, *p* < 0.001 in all analyses). Major developmental events are outlined in Fig. 3 (also see Table 4 for statistical results). All key embryonic features normally occurring after 2–4 days (velum, statocysts, left and right digestive diverticula, operculum, eyespots and protoconch) appeared approximately 24 h earlier under elevated seawater temperature compared with ambient conditions. Furthermore, whereas the development of embryos was completed within 4.78 ± 0.3 d (mean ± SD; range: 4.41–5.16 d) after oviposition in ambient temperature, veligers hatched after only 3.67 ± 0.34 d (mean ± SD; range: 3.25–4.16 d) in elevated temperature. Veligers were also significantly smaller in elevated temperature seawater when compared with ambient conditions. The mean lateral shell length and spiral height in ambient temperature were 94.44 ± 6.85 µm and 71.86 ± 5.78 µm, respectively, compared to 77.72 ± 5.82 µm (−18% compared to ambient conditions) and 57.14 ± 5.01 µm (−21% compared to ambient conditions) in elevated temperature seawater (Fig. 4). Paired *t*-tests found significant differences between treatments for both larval shell dimensions (lateral shell length: *t* = 10.184, *d*.*f* = 58, *p* < 0.001; spiral height: *t* = 10.534, *d*.*f* = 58, *p* < 0.001).

**Table 4:** Effects of temperature on embryonic development. Results of the mixed-model nested analysis of variance (ANOVA) for when 50% of embyros reached key embryonic features between ambient and elevated temperature conditions.

Embryonic variable	Among treatments			Within treatments	
	*d*.*f*	*F*	*P*	*F*	*P*
Velum	26	142.2	**<0.001**	2.349	0.137
Statocysts	26	98.67	**<0.001**	0.36	0.554
Digestive diverticula	26	258.5	**<0.001**	0.256	0.617
Operculum	26	279.9	**<0.001**	0.613	0.441
Eyespots	26	81.75	**<0.001**	0.242	0.627
Protoconch	26	96.51	**<0.001**	1.369	0.253
Hatching	26	82.42	**<0.001**	0.229	0.636

**Notes.**

Significant differences are marked with bold values. *d*.*f*, degrees of freedom.

## Discussion

The present study successfully documented the embryonic life history of *S. striatus* and revealed that the rate of appearance of some of its key features is controlled by the thermal environment. Like many other opisthobranchs and especially aplysiid species, its embryonic period is short (mean ± SD: 4.53 ± 0.2 d), but within the range for species with planktotrophic larvae (Hadfield & Switzer-Dunlap, 1984).

Before the onset of copulation, the pre-mating behavior (i.e., courtship) was characterized by multiple contacts between the two reproductive partners, possibly as a means of species recognition by chemoreception (Hamel & Mercier, 2006). Based on similar courtship interactions observed in other species (e.g., Karlsson & Haase, 2002), individuals initiating contact may acquire information on the sexual status of a potential mate or complete the final stages of gametogenesis (Hamel, Sargent & Mercier, 2008). As for the vast majority of opisthobranchs, *S. striatus* was found to cross-fertilize in the head-to-tail position, side by side (Hadfield & Switzer-Dunlap, 1984). The percentage of simultaneously reciprocal mating and subsequent egg-laying in our study was 47% (Table 1), falling within the wide array of mating patterns observed among opisthobranchs which vary from purely unilateral to partial and exclusive reciprocal mating (Anthes & Michiels, 2007). The mating type of *S. striatus* is typical of species having simultaneously reciprocal penial intromission and insemination in approximately half their mating sequences, such as for the well-described *Navanax inermis* (see Leonard & Lukowiak, 1985; Michiels, Raven-Yoo-Heufes & Kleine Brockmann, 2003). The interval between copulation and egg-laying, which is most likely due to the necessary time for activation of exogenous sperm, is slightly shorter (9.2 ± 3.42 h; Table 1) for our study species in comparison to the usual 12–24 h for other opisthobranchs (Hadfield & Switzer-Dunlap, 1984). The laying duration of an egg mass varies widely among opisthobranch species, from less than 1 h to more than 10 h (Hadfield & Switzer-Dunlap, 1984). Oviposition by *S. striatus* lasts approximately 5–15 min, depending on the length of the egg mass, similar to egg-laying duration observed for other aplysiids, such as *Aplysia californica* (Ferguson et al., 1989) and *Aplysia brasiliana* (Cobbs & Pinsker, 1982).

The number of embryos per egg capsule is variable across the Opisthobranchia (Todd, 1981; Hadfield & Switzer-Dunlap, 1984). While single encapsulation is prevalent and considered ancestral, deviations occur in almost all opisthobranch groups. In *S. striatus* the average ± SD of embryos in each egg capsule was 2.68 ± 0.76 (Table 2). This deviation from single encapsulation may be related to a large maternal body size, rather than being species-specific, as suggested by Mikkelsen (1996). As such, the fact that larger individuals were chosen for this study (mean length ± SD of 5.6 ± 0.73 cm; Table 1) may explain why there were more eggs in a single capsule.

The embryo size of *S. striatus* can be classified in the small-embryo (30–170 µm) group, based on the conclusions by Thompson (1967). The rapid larval development from small-sized embryos is typical of species developing into planktotrophic larvae (Goddard, 2004). The small amount of food stored inside the digestive tract of *S. striatus* is also typical of planktotrophic species, as lecithotrophic species contain much more stored food for embryonic development (Chester, 1999). The fast development of *S. striatus* results in a relatively short embryonic period, with an average of 4.5 d (Table 3). Although the developmental period from spawning to hatching varies among aplysiid species, *S. striatus* falls within this range which is generally <16 days (Lee, Kaang & Lee, 2014). Larval morphology at hatching was typical of planktotrophic opisthobranch veligers (Chia & Koss, 1978; Bickell & Kempf, 1983), including a notable type 1 shell (Thompson, 1961); yet shell length at hatching (mean ± SD of 94.44 ± 6.85 µm) is smaller than most other opisthobranch larvae that have been reared in the laboratory (Hadfield & Switzer-Dunlap, 1984).

Significant effects of temperature on developmental stages were found in agreement with findings that environmental conditions have major impacts on early developmental and reproductive stages of marine invertebrates (Gibson et al., 2011). In this study, embryos from egg masses in elevated temperature developed faster and hatched earlier (approximately 24 h earlier) (Fig. 3), but were smaller in size than those in ambient seawater (∼20% decrease in lateral shell length and spiral height) (Fig. 4). These results support other findings of faster embryonic development and reduced larval size under elevated seawater temperature conditions (EJ Armstrong , T Allen, M Beltrand, V Dubousquet, JH Stillman, SC Mills, 2012 & 2015, unpublished data), however, the results described herein detail the impacts on a wider range of embryonic stages, specifically early stages. This is in agreement with a phenomenon known as the temperature-size rule (Atkinson, 1994), and may be attributed, at least in part, to differences in seawater temperature having pervasive effects on oxygen consumption and transport within the egg mass (Moran & Woods, 2007). The decrease in hatching time under elevated temperature found here has been well established for many invertebrates (e.g., Cancino, Gallardo & Torres, 2003; Pechenik, Marsden & Pechenik, 2003; Watt & Aiken, 2003). On the other hand, if water temperature exceeds thermal thresholds, the ensuing thermal stress may result in a longer larval developmental rate, physiological stress, and eventual mortality (Cancino, Gallardo & Torres, 2003; Przeslawski, 2004). Accordingly, future work on *S. striatus* must elucidate the impacts of elevated seawater temperature on subsequent larval growth rate, development, survival, time until metamorphic competency, and metamorphic success rate. This data is crucial to assess whether the reduced larval size observed here may impair efficient food intake, increase vulnerability to predation and lead to reduced settlement success, ultimately reducing fitness and increasing mortality (e.g., Marshall & Steinberg, 2014).

In the case of *S. striatus*, it is important to note that tropical species such as this have developed in stable thermal environments, and thus may be especially sensitive to temperature increases deviating from the present prevailing conditions (Stillman, 2003; Tewksbury, Huey & Deutsch, 2008). Future work should also incorporate the use of an intermediate temperature (e.g., 30 °C) to demonstrate the effects of a slight temperature increase on embryonic growth rate and hatching size, thus discerning whether the upper-threshold projected temperature used in the current study (31 °C) is a possible “tipping point” for this species. Our results suggest that the ensuing potential decrease of this species’ fitness under predicted near-future ocean warming may have significant ecological ramifications, and potentially affect its important role in regulating toxic blooms of the cyanobacterium, *L. majuscula* (Thacker & Paul, 2004).

## Supplemental Information

10.7717/peerj.2956/supp-1 Data S1 Raw data

## Additional Information and Declarations

### Field Study Permissions

The following information was supplied relating to field study approvals (i.e., approving body and any reference numbers): Approval was granted from the institutional animal ethics committee at the CRIOBE research station for specimen collection and dissection (permit Number: 006725).

### Data Availability

The following information was supplied regarding data availability: The raw data has been supplied as a Supplementary File.
